# Recruitment results among families contacted for an obesity prevention intervention: the Obesity Prevention Tailored for Health Study

**DOI:** 10.1186/1745-6215-15-463

**Published:** 2014-11-27

**Authors:** Nirupa R Ghai, Kim D Reynolds, Anny H Xiang, Kimberly Massie, Sabrina Rosetti, Lyzette Blanco, Mayra P Martinez, Virginia P Quinn

**Affiliations:** Department of Research & Evaluation, Kaiser Permanente Southern California, 100 S. Los Robles, 2nd Floor, Pasadena, CA 91101 USA; School of Community and Global Health, Claremont Graduate University, 675 W. Foothill Blvd, Ste. 310, Claremont, CA 91711 USA

**Keywords:** Obesity prevention, Families, Children, Recruitment

## Abstract

**Background:**

Overweight and obesity are serious threats to health and increase healthcare utilization and costs. The Obesity Prevention Tailored for Health (OPT) study was designed to test the effectiveness of a family-based intervention targeting diet and physical activity. We describe the results of efforts to recruit parents and children enrolled in a large managed-care organization into the OPT study.

**Methods:**

Parents with 10- to 12-year-old children were randomly selected from the membership of Kaiser Permanente Southern California, a large integrated health plan, and contacted between June 2010 and November 2011. We describe recruitment outcomes and compare characteristics of parents and children who did and did not participate. Information was collected from calls with parents and through the administrative and electronic medical records of the health plan.

**Results:**

Of the 4,730 parents contacted, 16.1% expressed interest in participation (acceptors), 28.8% declined participation (refusers), 4.7% were ineligible, and, even after multiple attempts, we were unable to reach 50.4%. Slightly less than half of the acceptors (n = 361) were ultimately randomized to receive either the OPT program plus usual care or usual care alone (7.6% of all parents initially contacted). There were not any significant differences between acceptors who were or were not randomized. Overall, we found that acceptors were more likely to be female parents, have overweight/obese children, and higher utilization of outpatient visits by parents and children compared with refusers and those we were unable to reach. We found no differences in recruitment outcomes by body mass index or comorbidity score of the parents, level of physical activity of the parents and children, education of the parents, or household income.

**Conclusions:**

Recruiting parents and children into an obesity prevention program in a healthcare setting proved to be challenging and resource-intensive. Barriers and incentives for participation in obesity prevention programs need to be identified and addressed. Concern for the weight of their children may motivate parents to participate in family-based lifestyle interventions; however, the healthcare setting may be more relevant to weight-related treatment than to primary prevention.

**Trial registration:**

Trial Registration Number: ISRCTN06248443, 30 January 2014.

## Background

The prevalence of obesity in the US among children, adolescents and adults continues to be a serious public health concern. Between 1988 and 2010 the age-adjusted national prevalence of overweight and obesity among adults aged 20 years and older rose from 56.0% to 68.8% [1]. Among children, the prevalence of obesity increased from 7.2% to 12.1% in 2- to 5-year-olds, from 11.3% to 18.0% in 6- to 11-year-olds, and from 10.5% to 18.4% in those aged 12 to 19 years [1]. Overweight and obesity increases the risk for chronic health conditions in adults and children including hypertension, diabetes, dyslipidemia, and sleep apnea [2, 3]. Additionally, obese and overweight adults are more likely to suffer from heart disease, stroke, osteoarthritis, and some cancers [3]. In children, being overweight or obese increases the likelihood of asthma, joint problems, fatty liver disease, and social and psychological problems [2]. Importantly, compared to healthy-weight children, those who are overweight or obese are twice as likely to be overweight or obese as adults [4].

Physical inactivity, unhealthy eating patterns, and ethnic minority status are among the other factors associated with overweight and obesity in adults and children [5–8]. In addition, parental body mass index (BMI), parenting style, childhood behavioral problems, parental education, and household income are associated with excess weight in children [5–7]. Previous research has shown that family-based lifestyle interventions have been successful in reducing BMI in children [5, 9–30]. These studies highlight the role parents play in modeling healthy eating patterns and being physically active [5, 9–30]. Recruitment into these studies occurred in diverse settings including schools, primary care clinics [17, 29], hospitals [13, 21], outpatient referral clinic systems [16, 24–26] and specialty clinics [27, 28, 30]. Most of these studies were focused on treatment not prevention, and exclusively recruited children who were already overweight or obese [13, 16, 17, 21, 24, 25, 27–30]. To our knowledge, the only study to date that recruited children from a managed-care organization examined an obesity treatment intervention among female adolescents [31]. We believe the Obesity Prevention Tailored for Health (OPT) study is unique in recruiting parents and their children into a family-based obesity prevention intervention through a healthcare-managed organization. Given the serious health consequences of obesity and overweight, managed-care organizations with their emphasis on health promotion may be an especially relevant setting for obesity prevention. We describe the results of efforts to recruit parent–child pairs enrolled in a large managed-care organization for participation in an obesity prevention study.

### Obesity Prevention Tailored for Health

The OPT intervention study was designed and conducted by investigators from Claremont Graduate University, Kaiser Permanente Southern California (KPSC), the University of Southern California, and the University of Colorado at Denver. The protocol was reviewed and approved by the respective institutional review boards (IRBs) of the investigators for protection of the human subjects. The goal of the study was to test the effectiveness of a family-based intervention targeting four behaviors: increased consumption of fruits and vegetables; decreased consumption of saturated fat; increased physical activity; and decreased sedentary time. Additional information and advice were provided about portion sizes and consumption of sweetened beverages. The OPT intervention was delivered over 18 weeks to a parent and his/her 10- to 12-year-old child. To be eligible, the parent had to be a member of the KPSC health plan for at least 1 year and both the parent and child had to be an English speaker. A parent–child pair was excluded if the child had a major illness such as cancer, heart disease or diabetes, or was already receiving clinical treatment for obesity. The pair were randomly assigned to receive either usual care (physician advice and access to classes for nutrition and weight management) or usual care plus the OPT intervention.

The OPT intervention arm consisted of an hour-long in-person meeting between the parent–child pair and a health coach trained in motivational interviewing [32, 33]. Content of the counseling included educational information and printed materials describing the benefits of healthy eating and physical activity. To individually tailor the counseling, the coach assessed the goals of both the parents and children, and attitudes, confidence, and readiness about making changes in their diet and physical activity. With guidance from the coach, parents and children each selected their initial target behavior and created an individual action plan for the following 2 to 3 weeks. The session with the health coach was followed by five 20- to 30-minute telephone counseling calls with the parent delivered by trained motivational interviewers from the University of Colorado Cancer Center, Anschutz Medical Campus. In order to address each of the four target behaviors, over the course of the calls, counselors supported parents in creating three additional action plans for behavior change. In addition, the counselors mentored parents in guiding their children to develop action plans of their own. Both parents and children received four culturally tailored newsletters through the mail, two specific to diet and two specific to physical activity. The newsletters included structured family activities focused on the target behaviors.

## Methods

### Recruitment setting

KPSC is an integrated healthcare organization that provides comprehensive medical services to 3.6 million members. Individuals enroll in the health plan through their employer or the employer of a family member, individual plans, or state and federal programs such as CALPERS, Medi-Cal, and Medicare. The membership is socioeconomically diverse and broadly representative of the underlying population living in southern California [34]. The KPSC membership is 40% Hispanic, 37% white, non-Hispanic, 10% African-American, 10% Asian/Pacific Islander, and 3% other race/ethnicity. Seventeen percent of children aged 10 to 14 years in southern California receive healthcare through KPSC [35]. Over a third (37.1%) of the KPSC pediatric population is overweight and 19.4% is obese, with the highest prevalence among Hispanics [35]. The OPT study participants were recruited from the KPSC Downey Medical Center serving 300,000 patients in southeast Los Angeles County. The study was reviewed and approved by the IRBs for the protection of the human subjects at KPSC (IRB #4916), Claremont Graduate University (IRB #1064), University of Southern California (IRB #HS-07-00436) and University of Colorado at Denver (IRB #09–1145).

### Recruitment methods

Random samples of potentially eligible families were drawn on a regular basis from the membership files of the health plan between June 2010 and November 2011. Prior to contacting parents, KPSC recruitment staff notified the pediatricians of the children by email to prepare them for questions they could receive about the study and to provide them the opportunity to identify families who should not be contacted (for example, children undergoing clinical treatment for obesity or families in distress). Although the pediatricians were informed of the study, they did not participate in recruitment of families. We sent the parent who was the subscriber to the health plan an introductory letter with a fact sheet describing the study in detail. The letter advised parents that a member of the KPSC research staff would be calling them in the next 2 weeks to assess their eligibility and interest in participating in the study. Trained interviewers from KPSC made up to seven attempts to reach parents, calling on weekdays and weekends and at different hours of the day and evening. Interviewers left messages on answering machines for parents to call a toll free study number if they were interested in participating or had questions about the study. The KPSC interviewer requested permission to disclose the name and contact information of the parent to Claremont Graduate University research staff when eligible parents expressed interest in the study. We labeled these families “acceptors” and scheduled parents and their children for a 2-hour in-person appointment to obtain written informed consent from parents and assents from children, and baseline study measures which included a diet history questionnaire for parents and a survey about physical activity and diet for both the child and parent. At the completion of data collection, the parent–child pair was randomized to either the OPT or usual care study arms.

### Characteristics of participants and non-participants

To compare the characteristics of parents and children who did and did not participate in the OPT study and those we were unable to reach, we obtained information from the administrative and electronic medical records of the health plan. Demographic characteristics included gender, age at study contact date, race/ethnicity, and geocoded annual household income and education. Geocoded education was dichotomized at the neighborhood level into high school or less and some college or more.

We also extracted the length of health plan membership of the parents. Height and weight measures from clinical encounters were used to calculate BMI defined as weight in kilograms divided by the square of height in meters (kg/m^2^). In adults, BMI was categorized into: underweight (<18.5), healthy weight (18.5 to 24.9), overweight (25 to 29.9) and obese (≥30), based on the guidelines established by the National Institutes of Health [36]. Among children, BMI was calculated then percentile ranked according to gender-specific CDC growth pattern charts into the following categories: underweight (<5th percentile), healthy weight (5th percentile to <85th percentile), overweight (85th percentile to <95th percentile) and obese (≥95th percentile).

Shortly before the start of the study in 2009, KPSC initiated assessment of physical activity queried during clinical encounters [37]. We extracted reports of minutes of physical activity per week from visits closest to the date of study contact. Health conditions of the parents in the prior 3 years including hypertension (International Classification of Diseases-9 (ICD-9) : 401), hyperlipidemia (ICD-9: 272), and diabetes (ICD-9: 250 or a prescription for insulin or a laboratory value of hemoglobin A1c levels >7.5) were obtained from the electronic medical record. In addition, a modified weighted Charlson score based on seventeen categories of co-morbid diagnoses in the electronic records was calculated to characterize the burden of medical co-morbidities of the patients [38]. Finally, we assessed utilization of health services in the previous 3 years with counts of outpatient visits, health education classes attended, and flu vaccinations.

### Data analysis

Recruitment results are described with simple descriptive statistics. Bivariate analyses were conducted using 2 × 2 contingency tables for categorical data and 2-sided *t* tests for continuous variables. Characteristics of both parents and children were compared across the four recruitment outcome categories: accepted the study and randomized, accepted the study and not randomized, refused to participate, and not reached by phone after multiple attempts. Differences in frequencies were tested by chi-square tests, means by analysis of variance and medians by Kruskal-Wallis tests. All data was analyzed using SAS version 9.2 (SAS Institute, Inc., Cary, NC, USA).

## Results

**Figure 1:**
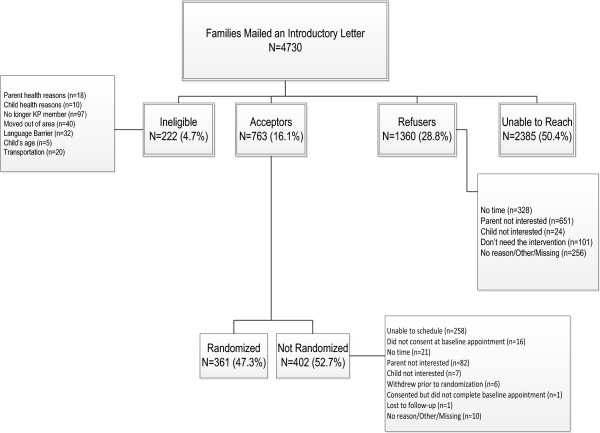
**CONSORT diagram for Obesity Prevention Tailored for Health OPT Study.** KPSC, Kaiser Permanente Southern California.

We sent letters introducing the OPT study to 4,730 randomly selected parents followed by telephone recruitment calls. Results of recruitment efforts found 222 parents or children (4.7% of families) were ineligible, 1,360 (28.8%) declined participation, and, despite multiple attempts and messages left on answering machines, we were unable to make contact with 2,385 parents (50.4%). We scheduled 763 (16.1%) “acceptors” for a baseline appointment. Of these parent–child pairs scheduled, 47.3% consented, completed baseline assessments and were randomized into either the OPT or usual care treatment arms (7.6% of all parents initially contacted). Reasons for non-participation are detailed in the recruitment CONSORT diagram (Figure 1). The most frequent reason given for declining participation at the recruitment call was lack of interest on the part of either the parent or child (49.6%). Nearly a quarter of the parents (23.1%) stated they did not have sufficient time to participate. The most important reason associated with acceptors not completing recruitment through randomization was their inability or unwillingness to keep or reschedule a baseline appointment.

Table 1 contrasts demographics across recruitment outcomes. We contacted similar proportions of male and female parents and children. On average parents were 40 years of age and children were 10.8 years of age. More than half of the parents were Hispanic (58.3%), half had more than a high school education, and the average median household income was approximately $55,000 per year. Parents who accepted the study whether or not they were ultimately randomized were more likely to be female (60.0% of randomized and 59.0% of those not randomized) compared with parents who refused participation (47.7%) or who were not reached (45.1%). Similarly, Hispanic parents comprised a larger proportion of acceptors than they did for other recruitment outcomes. Randomized acceptors and those who refused the study had slightly higher annual median household incomes compared to acceptors not randomized and those we were unable to reach.

**Table 1:** **Demographic characteristics of parents and children by recruitment outcomes**

	Accepted^a^and randomized (N = 361)		Accepted^a^and not randomized (N = 402)		Refused (N = 1,360)		Unable to reach (N = 2,385)		Total (N = 4,508)^b^	
	n	(%)	n	(%)	n	(%)	n	(%)	n	(%)
***Parents***										
Gender***										
Female	216	(60.0)	237	(59.0)	649	(47.7)	1076	(45.1)	2178	(48.0)
Male	145	(40.0)	165	(41.0)	711	(52.3)	1309	(54.9)	2330	(52.0)
Age (years) at study contact***										
Mean (SD)	39.9	(6.3)	40.1	(6.8)	41.0	(7.6)	39.5	(6.7)	40.0	(7.0)
Race/ethnicity***										
White non-Hispanic	42	(11.6)	24	(6.0)	131	(9.6)	151	(6.3)	348	(7.7)
African American/Black	42	(11.6)	74	(18.4)	191	(14.1)	369	(15.5)	676	(15.0)
Hispanic	235	(65.1)	255	(63.4)	741	(54.5)	1396	(58.5)	2627	(58.3)
Other non-Hispanic	21	(5.8)	19	(4.7)	158	(11.6)	197	(8.3)	395	(8.8)
Unknown	21	(5.8)	30	(7.5)	138	(10.2)	272	(11.4)	461	(10.2)
Missing	0	(0.0)	0	(0.0)	1	(0.1)	0	(0.0)	1	(0.0)
Education***										
≤ High school	187	(51.8)	200	(49.8)	770	(56.6)	1157	(48.5)	2314	(49.7)
> High school	174	(48.2)	202	(50.3)	590	(43.4)	1228	(51.5)	2194	(51.3)
Median household income***										
Median (interquartile range)	$56,574 ($29,962)		$54,130 ($27,067)		$58,761 ($26,555)		$54,490 ($26,779)		$55,497 ($27,092)	
***Child***										
**Gender**										
Female	177	(49.0)	197	(49.0)	700	(51.5)	1176	(49.3)	2250	(49.9)
Male	184	(51.0)	205	(51.0)	659	(48.5)	1209	(50.7)	2257	(50.1)
**Age (years) at study contact**										
Mean (SD)	10.8	(0.8)	10.8	(0.8)	10.8	(0.9)	10.8	(0.8)	10.8	(0.8)

^a^Parents from Kaiser Permanente Southern California who accepted offer of study participation and gave verbal consent to disclose contact information to Claremont Graduate University for consent and randomization. ^b^Excludes 222 families not eligible for participation. ***P < 0.0001 testing for differences among the 4 groups.

Table 2 describes clinical characteristics and utilization of health plan services by recruitment outcome status of the participants and non-participants. Overall, three-quarters of the parents and over 40% of children contacted for study participation were overweight or obese. Yet, in the 3 years prior to study contact, few parents attended health plan education classes for weight management. Forty-three percent of adults reported no regular physical activity and 29.2% of children reported 3 hours of activity or less a week. Most parents had no comorbid conditions (Charlson score of 0). On average, parents had maintained health plan membership for 10 years. Over half of parents (59.6%) and 39.2% of children had seven or more outpatient visits in the 3 years prior to study contact. Despite these medical encounters, over two-thirds of parents and 58.8% of children had not received flu vaccinations during that 3-year period.

**Table 2:** **Clinical characteristics of parents and children by recruitment outcomes**

	Accepted^a^and randomized (N = 361)		Accepted^a^and not randomized (N = 402)		Refused (N = 1,360)		Unable to reach (N = 2,385)		Total (N = 4,508)^b^	
	n	(%)	n	(%)	n	(%)	n	(%)	n	(%)
***Parents***										
**BMI category (kg/m** ^**2**^ **)** ^**c,**^ ***										
Mean (SD)	30.9	(6.5)	31.3	(7.0)	29.7	(5.8)	31.3	(6.6)	30.8	(6.4)
Underweight (<18.5)	18	(5.0)	18	(4.5)	121	(8.9)	320	(13.4)	477	(10.6)
Healthy weight (18.5-24.9)	55	(15.2)	62	(15.4)	245	(18.0)	288	(12.1)	650	(14.4)
Overweight (25-29.9)	127	(35.2)	123	(30.6)	462	(34.0)	699	(29.3)	1,411	(31.3)
Obese (30+)	161	(44.6)	199	(49.5)	532	(39.1)	1,078	(45.2)	1,970	(43.7)
**Physical activity levels (minutes/week)** ^**c**^										
Median (interquartile range)	60.0	(150.0)	60.0	(150.0)	60.0	(150.0)	60.0	(150.0)	60.0	(150.0)
0	116	(39.7)	151	(45.9)	428	(42.3)	737	(43.9)	1432	(43.2)
1-149	97	(33.2)	88	(26.8)	297	(29.4)	512	(30.5)	994	(30.0)
150	79	(27.1)	90	(27.3)	287	(28.4)	430	(25.6)	886	(26.8)
Missing	69		73		348		706		1,196	
Length of health plan membership (years)*										
Median (interquartile range)	10.1	(9.3)	9.0	(9.6)	9.6	(10.1)	8.8	(9.4)	9.2	(9.6)
**Number of outpatient visits** ^**d**,^ ***										
Median (interquartile range)	11.0	(16.0)	11.0	(13.0)	9.0	(13.0)	8.0	(11.0)	8.0	(12.0)
0	4	(1.1)	2	(0.5)	50	(3.7)	151	(6.3)	207	(4.6)
1-3	44	(12.2)	53	(13.2)	205	(15.1)	479	(20.1)	781	(17.3)
4-6	60	(16.6)	67	(16.7)	264	(19.4)	440	(18.5)	831	(18.4)
7+	253	(70.1)	280	(70.0)	840	(61.8)	1,315	(55.1)	2,688	(59.6)
Missing	0		0		0		0		1	
**Number of flu vaccines** ^**d**,^ ***										
Median (interquartile range)	0.0	(1.0)	0.0	(1.0)	0.0	(1.0)	0.0	(1.0)	0.0	(1.0)
0	207	(57.3)	260	(64.7)	927	(68.2)	1,686	(70.7)	3,080	(68.3)
1	79	(21.9)	86	(21.4)	214	(15.7)	398	(16.7)	777	(17.2)
2	37	(10.3)	31	(7.7)	102	(7.5)	153	(6.4)	323	(7.2)
3+	38	(10.5)	25	(6.2)	117	(8.6)	148	(6.2)	328	(7.3)
Missing	0		0		0		0		0	
**Charlson weighted score** ^**e**^										
0	313	(86.7)	344	(85.6)	1,168	(86.0)	2,042	(85.6)	3,867	(85.8)
1+	48	(13.3)	58	(14.4)	191	(14.0)	343	(14.4)	640	(14.2)
Missing	0		0		0		0		1	
***Health education classes***										
**Bariatric surgery class** ^**f,**^ ***										
Yes	15	(4.2)	16	(4.0)	22	(1.6)	44	(1.8)	97	(2.2)
No	346	(95.8)	386	(96.0)	1,338	(98.4)	2,341	(98.2)	4,411	(97.8)
Missing	0		0		0		0		0	
**Any nutrition class** ^**f,**^ *										
Yes	10	(2.8)	9	(2.2)	11	(0.8)	22	(0.9)	52	(1.2)
No	351	(97.2)	393	(97.8)	1,349	(99.2)	2,363	(99.1)	4,456	(98.8)
Missing	0		0		0		0		0	
**Weight management class** ^**f**^										
Yes	11	(3.1)	7	(1.7)	20	(1.5)	26	(1.1)	64	(1.4)
No	350	(96.9)	395	(98.3)	1,340	(98.5)	2,359	(98.9)	4,444	(98.6)
Missing	0		0		0		0		0	
***Children***										
**BMI category (percentile)** ^**c,**^ ***										
Mean (SD)	76.2	(26.6)	75.7	(27.0)	68.5	(29.7)	72.9	(28.1)	72.1	(28.5)
Underweight (<5)	32	(8.9)	40	(10.0)	201	(14.8)	406	(17.0)	679	(16.1)
Healthy weight (5- < 85)	144	(39.9)	161	(40.0)	630	(46.3)	982	(41.2)	1,917	(42.5)
Overweight (85- < 95)	75	(20.8)	76	(18.9)	263	(19.3)	407	(17.1)	821	(18.2)
Obese (≥95)	110	(30.4)	125	(31.1)	266	(19.6)	590	(24.7)	1,091	(24.2)
**Physical activity levels (minutes/week)** ^**c,**^ ***										
Median (interquartile range)	180.0	(200.0)	240.0	(180.0)	240.0	(150.0)	180.0	(200.0)	200.0	(180.0)
0	19	(10.9)	24	(12.8)	68	(10.9)	148	(14.6)	259	(12.9)
1-149	33	(18.9)	29	(15.4)	87	(13.9)	177	(17.5)	326	(16.3)
150-279	60	(34.3)	52	(27.7)	175	(28.0)	307	(30.3)	594	(29.7)
280-359	40	(22.9)	52	(27.7)	158	(25.3)	222	(21.9)	472	(23.6)
360+	23	(13.1)	31	(16.5)	136	(21.8)	160	(15.8)	350	(17.5)
Missing	186		214		736		1,371		2,507	
**Number of outpatient visits** ^**d**,^ ***										
Median (interquartile range)	7.0	(8.0)	6.0	(7.0)	5.0	(6.0)	5.0	(6.0)	5.0	(6.0)
0	7	(1.9)	13	(3.2)	66	(4.9)	152	(6.4)	238	(5.3)
1-3	79	(21.9)	99	(24.6)	354	(26.1)	715	(30.0)	1,247	(27.7)
4-6	89	(24.7)	103	(25.6)	400	(29.4)	662	(27.7)	1,254	(27.8)
7+	186	(51.5)	187	(46.5)	539	(39.7)	856	(35.9)	1,768	(39.2)
Missing	0		0		0		0		1	
**Number of flu vaccines** ^**d**,^ **										
Median (interquartile range)	0.0	(1.0)	0.0	(1.0)	0.0	(1.0)	0.0	(1.0)	0.0	(1.0)
0	187	(51.8)	229	(57.0)	786	(57.8)	1,480	(62.1)	2,682	(59.5)
1	93	(25.8)	90	(22.4)	294	(21.6)	489	(20.5)	966	(21.4)
2	40	(11.0)	49	(12.2)	138	(10.2)	226	(9.4)	453	(10.1)
3+	41	(11.4)	34	(8.4)	142	(10.4)	190	(8.0)	407	(9.0)
Missing	0		0		0		0		0	

^a^Parents from Kaiser Permanente Southern California who accepted offer of study participation and gave verbal consent to disclose contact information to Claremont Graduate University for recruitment and randomization. ^b^Excludes 222 families not eligible for participation. ^c^Body mass index (BMI) and physical activity assessment from clinical encounter within 2 years closest to study contact date. ^d^Within 3 years of study contact date. ^e^Within 1 year before contact date. ^f^Class within 2 years before contact date up to 2 years after contact date. *P < 0.05; **P < 0.001; ***P < 0.0001 testing for differences among the 4 groups.

There were a few noteworthy differences in recruitment outcomes by clinical characteristics. Acceptors were more likely to have had a child who was overweight or obese compared with refusers or those we were unable to reach (50.6% versus 38.9% and 41.8%, respectively). Among acceptors, parents and children had more outpatient visits and more flu immunizations especially compared with families we were unable to reach (Table 2).

## Discussion

Overweight and obesity are associated with serious health outcomes, and, in 2013, obesity was recognized as a chronic disease by the American Medical Association [39]. Healthcare settings have the potential to prevent obesity-related illness by identifying and intervening with at-risk adults and children. To take advantage of this opportunity, the OPT study recruited parents with their 10- to 12-year-old children into a family-based obesity prevention intervention from the membership of a large managed-care organization. Of the 4,730 families we contacted for the study, 16.1% agreed to participate and 7.6% were ultimately randomized to the OPT program plus usual care or usual care alone. Female parent, overweight/obesity among children, and utilization of outpatient visits of the parents and children were the most noteworthy characteristics of families who participated in the study. We found no differences in recruitment outcomes by BMI or comorbidity score of the parents, level of physical activity of the parents and children, education of the parents, or household income.

One of the Healthy People 2020 goals is to reduce the percentage of obesity in children, adolescents and adults by 10% [40]. The prevalence of obesity among adults and children contacted for participation in this study was even higher than national estimates. Physical activity is critical for weight management and loss [41]. The US physical activity guidelines recommend 150 minutes of moderate-intensity aerobic activity or 75 minutes of vigorous activity every week for adults [42]. Of concern, over 40% of parents identified for recruitment reported being physically inactive (0 minutes of physical activity per week) when assessed at medical encounters prior to the start of the study. The national guidelines for children and adolescents recommend 60 minutes or more of physical activity every day [42]. Among children contacted for the study, 59% reported 280 minutes or less of physical activity a week. Similar to national reports [43, 44], few adults and children contacted for this study met the national recommendations for physical activity.

Despite the obvious need for overweight and obesity prevention, only one in six families contacted by staff participated in the study. Half of the families who declined to participate when reached by telephone stated they were not interested. Similarly, few parents had utilized nutrition and weight-related programs available through the health plan prior to study contact. Previous research has suggested lower participation in weight-related programs is due to lack of time, lack of resources, perceptions related to weight status, as well as perceptions towards children participating in weight loss programs [45]. Although we attempted to minimize participant burden, a large proportion of parents initially reported they did not have time for the study or intervention program and, even after initially expressing interest, many families were unavailable for keeping or rescheduling their baseline study visit.

We found parents were more likely to participate in the OPT study when their children, but not themselves, were overweight or obese. These parents may have been focused solely on seeking treatment for their children. However, the obesogenic environment in the US promotes increased consumption of unhealthy food and decreased physical activity putting all adults and children at risk for overweight and obesity. Lower participation among families with healthy weight children may highlight the need for greater emphasis on obesity prevention in families in healthcare and in community settings. Alternatively, concern for weight of their children may serve to motivate overweight parents to participate in family-based treatment interventions.

We found parents and children with more outpatient visits and higher utilization of flu vaccination were more likely to express interest in the study, suggesting a more proactive or preventive health orientation in these families [40]. On the other hand, outpatient visits offer the opportunity for provider counseling about the dangers of overweight and obesity, as well as the importance of healthy eating and physical activity, and may have motivated parents to seek help through the intervention of the study.

Referrals from pediatricians and targeted mailings have been found to be successful recruitment strategies for pediatric obesity research among families [46]. In our study, we contacted all parents by letter and attempted follow-up phone calls. Although we informed parents that the pediatrician of their children had been notified about the study, we did not actively engage the physicians in our recruitment efforts, perhaps missing an opportunity to improve family participation in the study.

Even with multiple attempts on varying days of the weeks and times of day, we were unable to reach half the parents initially identified for recruitment by phone. Families we were unable to reach had lower utilization of healthcare services including flu immunizations than did families who participated in the OPT program suggesting lower levels of engagement with prevention and healthcare services. We attempted to recruit all families randomly selected from the membership of a managed-care organization without regard to weight, diet, physical activity and other lifestyle factors, or motivation to improve diet and physical activity. Although the percent of families recruited into the study was relatively low, it is reassuring that we were able to identify few and only moderate differences between participants and non-participants. Further, as the KPSC membership is representative of the population living in southern California, [34] study results may generalize to families with diverse socio-demographic characteristics.

Additional strengths of this study include our ability to obtain socio-demographic and clinical information for participants and non-participants alike, and use of a Microsoft Access database to accurately track recruitment results and record reasons for non-participation. Our results can inform recruitment planning efforts for population-based studies including the extent of resources necessary to empanel study samples. They highlight the need for more intensive outreach to families who are less engaged with preventive services. Finally, they can provide guidance for dissemination and implementation of positive program results underscoring the need to motivate, educate, and, perhaps, incentivize participation in obesity prevention programs. Study limitations include incomplete implementation of the clinical physical activity assessment at the time recruitment began resulting in missing data, especially for children. Further, physical activity assessments were subject to the potential biases associated with self-reports. Finally, we were unable to determine the eligibility, level of interest, and reasons for not participating among parents we were unable to contact by telephone.

## Conclusions

Recruiting families into an obesity prevention program in a healthcare setting proved to be challenging and resource-intensive. Barriers to, and incentives for, participation in obesity prevention programs need to be identified and addressed before the potential of the healthcare setting for intervention can be realized. Concern for the weight of their children may motivate parents to participate in family-based lifestyle interventions; however, the healthcare setting may be more relevant to weight-related treatment than to primary prevention.

## Abbreviations

BMI: body mass index
IRB: institutional review board
KPSC: Kaiser Permanente Southern California
OPT: Obesity Prevention Tailored for Health.
